# Editorial

**Published:** 1857-12

**Authors:** 


### Editorial.

### DUES TO THE REGISTER—A PROPOSITION.

We send out bills with the present number of the Register to all those in arrears for Vols. 10 and 11, and hope they will, without farther ceremony, send us the amount.

We owe the printer more money than we have got, and we fear more than we will have for a while; and we do think that a year and a half is long enough to wait for advance payment.

Some of our subscribers have been very prompt in the payment of their subscription; others have been just as *prompt* in non-payment. Come, dear sirs, about five hundred of you, send us six dollars each, and it will diminish the length of our *phiz* wonderfully.

To those who will forward us five dollars for Vols. 11 and 12 before the 1st of January, 1858, we will give a full receipt for the two volumes. Those to whom twenty per cent. is an object, we expect to hear from.

If we have sent bills by mistake to any who have paid, they will please excuse, and notify us of the fact, as mistakes will sometimes occur under the closest attention.

### FUND FOR THE PROMOTION OF DENTAL SCIENCE.

Some of the professional brethren, and especially those who have contributed to the enterprise, may desire to know what progress has been made by the committee which was appointed to take charge of the scientific investigations, a series of which it was proposed to begin, at the late Convention.

We would just say that the committee, according to the spirit of the instructions, issued four thousand circulars, setting forth the aims and objects of the enterprise as fully as possible in so small a compass. These have been sent abroad to the profession of the United States, so far as its members could be reached by the most extensive lists. The responses to these have been few in number; but we are not without encouragement. The most of these responses are from persons not heard from before on the subject, and they all speak in the highest terms of the enterprise.

From very few of the early advocates have we received anything; but we are warranted in saying that they all stand ready, upon a moment's notice, to render material aid.

The committee has done but little as yet toward setting an operator to work, from the fact that there is not sufficient funds to warrant the employment of any one; and the members of the committee do not feel like involv-ng themselves personally, without some assurance that the bills will be met.

If the profession felt the interest in the subject that we think it demands, there would very soon be an overflowing fund; and certainly a better investment could not be made.

Although no one is yet put to work. the committee has decided upon a person, and obtained his consent to conduct the investigations and experiments. We hope that all who intend to give this enterprise their aid will do so at once, and delay no longer.

The work may just as well be going on at once as not, for it *must* ultimately *go*. We intend within a few months to publish the names of all contributors to this fund. T.

---

### EXPOSED DENTAL PULP.

We would call attention to the resolution of the Dental Convention of Northern Ohio on the subject of treating and filling over-exposed pulps. It is all-important to have statistics. No man can afford to be in error on a subject so important. We hope those who were not at the Convention will also adopt the suggestion of the resolution. And we would also remark that it is not necessary, in all cases, to wait till next meeting to make a report. We would be happy to report the results of efforts in this direction in our March number. The same cases, if desirable, can still be reported at the Convention, as material for a statistical table. The age, constitution, and temperament of the patient should be reported, as well as the minutiæ of the treatment and its results. Since the Cleveland meeting, we have had the opportunity to observe the result of treatment in one of our cases, which we may as well report in this connection:

Case.—Mr. B., aged 25, nervous-sanguine temperament, general health good, called to have his teeth examined, October 14th, 1857. In excavating a large cavity on the anterior approximal surface of the right first bicuspid of the upper denture, I exposed and wounded the pulp, which bled freely at the time. I wiped out the cavity, applied chloroform to it, touched the wounded point of the pulp with creosote, dried the cavity, and filled it with gutta percha. The tooth gave no pain.

On the 18th of November I removed the gutta percha, and found the tooth alive and healthy, and the pulp perfectly protected by a firm, bony covering. I now filled it with gold without any difficulty. A week later the tooth was still doing well. W.

---

### COMMENCEMENT EXERCISES.

The Commencement exercises of the Ohio College of Dental Surgery will take place in the lecture-room of the College, on Wednesday evening, February 17th, 1857. This occurring during the meeting of the Mississippi Valley Association, we hope our brethren from abroad will feel sufficient interest to induce them to favor us with their presence.

### "QUI SAPIT."

A correspondent of the Boston Medical and Surgical Journal seems to be sorely exercised over the fact that the American Dental Convention did not discuss the early history of the dental profession and its relation to the medical. Well, we suppose the Convention wisely came to the conclusion that those present were already somewhat posted up on the profession's early history, and felt more concern for its present and *future*, than for its past history. And as to its relation to the medical profession, having declared independence, the dental profession cares less for its *relations*, even though they be its parents, than formerly. But to be serious, these subjects have been discussed, both by tongue and pen, till they are worse than thread-bare. Their discussion at the Boston meeting would have been an unmitigated bore to the entire profession; and we are surprised that "Qui Sapit" should think otherwise.

---

### DENTAL CONVENTION OF NORTHERN OHIO.

We had the pleasure of attending the Convention above named, and most certainly feel that it was good to be there. The whole-souled heartiness with which everything was conducted was truly refreshing. We need not say that the report in this number fails to do the brethren there justice. The subjects for discussion not being announced till the meeting, the Convention labored under quite a disadvantage. We are sorry that we can not give a fuller and more faithful report of their discussions; but we trust the good-will may be accepted for the deed. In their modesty, they had concluded that their remarks would not be worth reporting, and had, therefore, made no provisions for a report. Arriving at the hour appointed, we volunteered our services, and we know that our readers will thank us for snatching from oblivion, even as much as we did of their discussions.

---

### THE OHIO DENTAL COLLEGE ASSOCIATION

Will meet at the College on Monday, February 15th, at 2 o'clock, P. M. A full meeting is very desirable. When members can attend this meeting, the Commencement exercises of their own College, and the meeting of the Mississippi Valley Association, all in the same week, and still, in most cases, have time to reach home, it would seem that the inducement to spend a week would be too tempting to resist. Yet many excuse themselves by saying that they are too busy, or that their patrons think hard if they are absent. If they really are busy, they can *afford* to go to dental meetings; and as to the latter excuse, their patrons will never think hard, if they know why they are absent, but will, on the contrary, hold them responsible for neglecting such opportunities for professional improvement. The public mind is becoming awakened, and is beginning to appreciate those members of the profession who are endeavoring to keep up with the times.

### MISSISSIPPI VALLEY ASSOCIATION.

This association, now the oldest dental society in existence, stands adjourned to meet on Wednesday, the 17th of February, at 10 o'clock, A. M. We invite special attention to the report of the Executive Committee, which is found on another page. We hope members, and others who may participate in the discussions will systematize their ideas;—especially on their favorite topics, so that the meeting may be, in all respects, profitable, and worthy of the pioneer society of the West.

---

### SWALLOWING ARTIFICIAL TEETH.

The following we clip from a fragment of a number of the New York Tribune, of which we know not the date. The termination of the case is as might be expected from such an accident; yet it is different from that of a case which occurred in this city in the hands of W. H. Mussey, M. D., which, we believe, was reported in the Register of 1854. In that case the plate, with the teeth attached, traversed the entire intestinal canal, without serious injury to the patient. A similar accident, with like happy termination, befel a servant girl, in the city of London, who was at the time an inmate of the house of Robt. LeBlond, Esq., now of this city.

A SINGULAR DEATH—KILLED BY SWALLOWING FALSE TEETH.—On the 10th inst., a man named Duncan McDougall was admitted to Bellevue Hospital, suffering, as he stated, by having swallowed some false teeth. He was placed under the care of Dr. Briest, one of the physicians attached to that institution; but the patient continued to fail, and died on Wednesday last. Coroner Connery held an inquest on the body, when Dr. E. B. Briest made a *post-mortem* examination of the body, which resulted in his finding that a foreign body, viz: two artificial teeth, and a gold plate on which they had been set, had lodged in the esophagus, about two inches above the cardiac orifice of the stomach, opposite the heart, thereby causing ulceration through into the pericardium, producing inflammation and obstruction of this membrane, and causing death. Under what circumstances the deceased swallowed the teeth and plate which caused death, was not developed before the Coroner. It is believed, however, that they slipped down his throat in the night-time while he was asleep. The Jury rendered a verdict that Duncan McDougall, the deceased, came to his death "by ulceration through the esophagus and pericardium, through the lodgment of a foreign body." The deceased was thirty-four years of age, and a native of Scotland. The residence of the deceased was not ascertained.—*N. Y. Tribune.*

---

DIED,—October 24th, 1857, Dr. William Grimes, Surgeon Dentist, of Richmond, Indiana, aged 28 years.

Dr. Grimes was a man of good attainments, and of great promise—such a man as his friends, the profession, and the public are never ready to spare; yet he is cut down in the bloom of youth. His disease was consnmption. Truly God's ways are not our ways.

